# Proteomics data on MAP Kinase Kinase 3 knock out bone marrow derived macrophages exposed to cigarette smoke extract

**DOI:** 10.1016/j.dib.2017.05.049

**Published:** 2017-06-07

**Authors:** Roshni Srivastava, Praveen Mannam, Navin Rauniyar, TuKiet T. Lam, Ruiyan Luo, Patty J. Lee, Anup Srivastava

**Affiliations:** a Department of Internal Medicine, Yale University School of Medicine, USA; b MS & Proteomics Resource at Yale University, WM Keck Foundation Biotechnology Resource Laboratory, Department of Molecular Biophysics and Biochemistry, New Haven, CT, USA; c Department of Epidemiology & Biostatistics, School of Public Health, Georgia State University, Atlanta, GA, USA; d Division of Translational and Regenerative Medicine, Internal Medicine, University of Arizona, Tucson, AZ, USA

**Keywords:** MKK3, Cigarette smoke, Inflammation, Proteomics, iTRAQ®

## Abstract

This data article reports changes in the phosphoproteome and total proteome of cigarette smoke extract (CSE) exposed WT and MAP Kinase Kinase 3 knock out (MKK3^−/−^) bone marrow derived macrophages (BMDM). The dataset generated is helpful for understanding the mechanism of CSE induced inflammation and the role of MAP kinase signaling pathway. The cellular proteins were labeled with isobaric tags for relative and absolute quantitation (iTRAQ®) reagents and analyzed by LC-MS/MS. The standard workflow module for iTRAQ® quantification within the Proteome Discoverer was utilized for the data analysis. Ingenuity Pathway Analysis (IPA) software and Reactome was used to identify enriched canonical pathways and molecular networks (Mannam et al., 2016) [1]. All the associated mass spectrometry data has been deposited in the Yale Protein Expression Database (YPED) with the web-link to the data: http://yped.med.yale.edu/repository/ViewSeriesMenu.do;jsessionid=6A5CB07543D8B529FAE8C3FCFE29471D?series_id=5044&series_name=MMK3+Deletion+in+MEFs

## **Specifications Table**

**Table:** 

Subject area	*Cell Biology and Kinase signaling*
More specific subject area	*MAP kinase signaling*
Type of data	*Table and workflow*
How data was acquired	*LC-MS/MS on a Q Exactive Plus mass spectrometer (ThermoFisher Scientific, San Jose, CA) interfaced with a nanoACQUITY UPLC System (Waters, Milford, MA)*
Data format	*The standard workflow module for iTRAQ(R) 8-plex quantification within the Proteome Discoverer was utilized for the analysis. Peptide spectrum matches (PSMs) were verified based on q-values set to 1% false discovery rate (FDR) using the Percolator module*
Experimental factors	*Effect of cigarette smoke on the proteome of mouse bone marrow derived macrophages and effect of MKK3 deletion*
Experimental features	*Baseline difference in MKK3 knockout and wild-type mouse bone marrow derived macrophages and their response to cigarette smoke*
Data source location	*New Haven, CT, USA.*
Data accessibility	*Data available within this article and at*http://yped.med.yale.edu/repository/ViewSeriesMenu.do;jsessionid=6A5CB07543D8B529FAE8C3FCFE29471D?series_id=5044&series_name=MMK3+Deletion+in+MEFs

## V**alue of the data**

• Proteomic characterization of the effect of MKK3 deletion in bone marrow derived macrophages (BMDM), which provides data on MAP kinase signaling targets [2], [3], [4], [5], [6], [7].
• The BMDM proteomics data (total and phospho) has been presented which can be used as a reference for other inflammatory signaling molecules in MAP kinase pathway.
• The major canonical pathways affected after cigarette smoke in WT and MKK3 deleted BMDM are presented and can be utilized to interrogate the role of MAP kinases in other signaling schemes in cigarette smoke induced injury.

## 1 Data

**Fig. 1:**
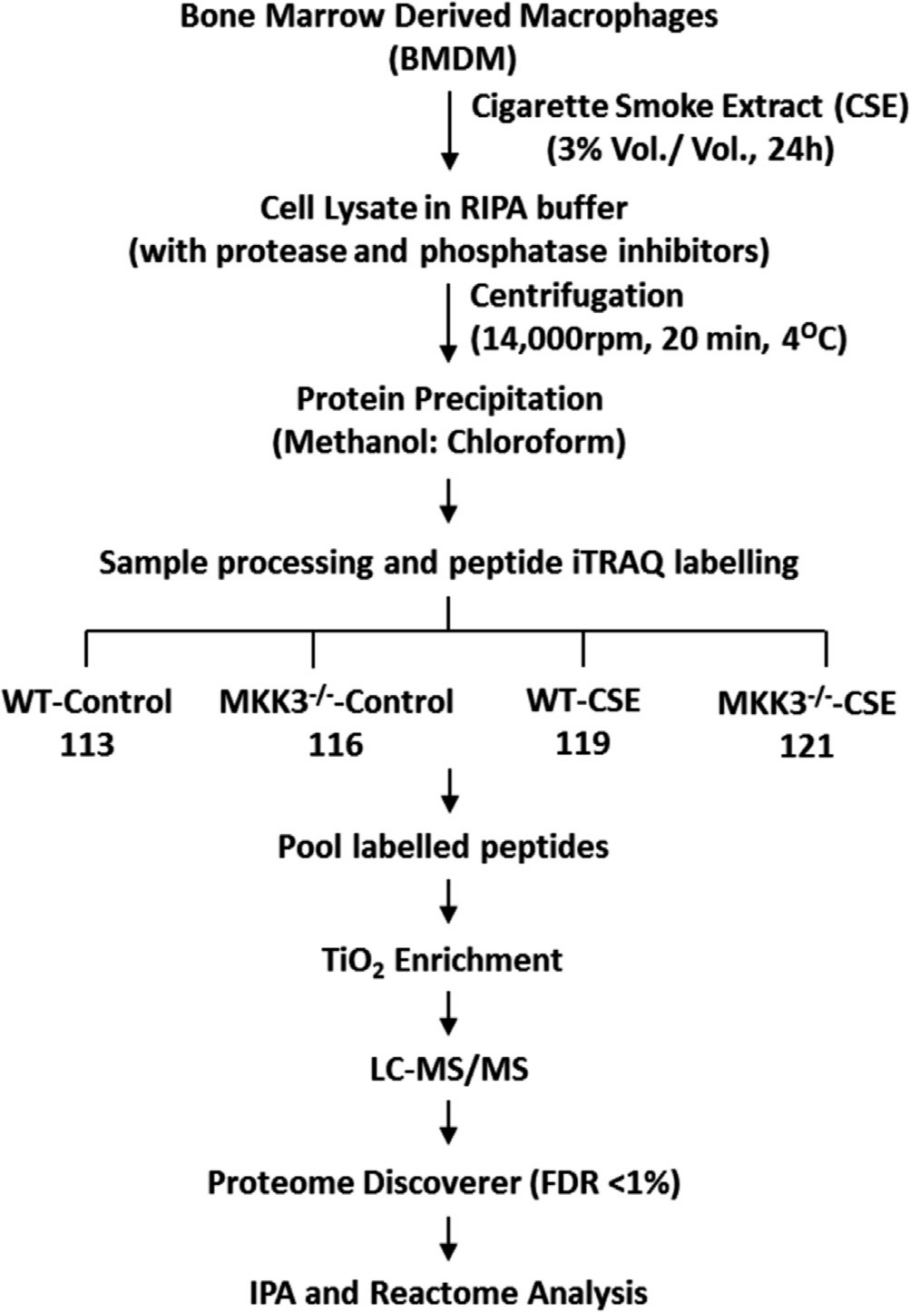
The workflow scheme of iTRAQ® data generation.

In this paper, we provide proteomics data generated before and after cigarette smoke extract (CSE) exposed BMDM isolated from MKK3^−/−^ and WT mice. The workflow is presented as Fig. 1. The data was analyzed using REACTOME, pathway search engine. The iTRAQ® quantitative proteomics data of MKK3^−/−^/WT BMDM at baseline showed enrichment of many canonical pathways, presented in Table 1. The comparison of WT and MKK3^−/−^ BMDM responses after CSE is presented in Table 2. Using QIAGEN׳s Ingenuity® Pathway Analysis (IPA®, QIAGEN Redwood City) the analysis of the data reflects changes in the molecules upregulated by MKK3 with the threshold criteria of at least 4 proteins affected (Table 3).

**Table 1:** Affected pathways in MKK3^−/−^ BMDM.

**#**	**Pathway name**	**#Entities found**	**Entities ratio**	**Entities pValue**	**Entities FDR**	**#Reactions found**
1	Neutrophil degranulation	57	0.045862794	1.27E−13	1.63E−10	10
2	L13a-mediated translational silencing of Ceruloplasmin expression	23	0.010701319	2.51E−10	1.07E−07	3
3	GTP hydrolysis and joining of the 60S ribosomal subunit	24	0.010796866	6.64E−10	1.71E−07	3
4	Formation of a pool of free 40S subunits	21	0.009745844	1.75E−09	3.21E−07	2
5	Cap-dependent Translation Initiation	24	0.011465698	7.56E−09	9.83E−07	18
6	Eukaryotic Translation Initiation	24	0.011465698	8.48E−09	9.83E−07	21
7	Ribosomal scanning and start codon recognition	15	0.005637302	3.30E−08	3.23E−06	2
8	Formation of the ternary complex, and subsequently, the 43S complex	13	0.004968469	1.87E−07	1.59E−05	3
9	Nonsense Mediated Decay (NMD) independent of the Exon Junction Complex	18	0.009172559	4.07E−07	3.26E−05	1
10	Translation	26	0.015096503	4.97E−07	3.53E−05	35

The proteomic data at baseline conditions was utilized to generate affected pathways using REACTOME. The data is generated from 3 biological replicates.

**Table 2:** Affected pathways in MKK3^−/−^ BMDM after cigarette smoke exposure (CSE).

**#**	**Pathway name**	**#Entities found**	**Entities ratio**	**Entities *p* Value**	**Entities FDR**	**#Reactions found**
1	Neutrophil degranulation	21	0.045862794	1.47E−06	0.001291321	9
2	Glucose metabolism	7	0.007739346	2.59E−04	0.113837907	13
3	Glycogen breakdown (glycogenolysis)	3	0.001433212	0.001054291	0.205181824	7
4	Glycolysis	4	0.003248615	0.001092518	0.205181824	4
5	Ribosomal scanning and start codon recognition	5	0.005637302	0.001405355	0.205181824	2
6	Sphingolipid metabolism	7	0.008503726	0.001933118	0.2416397	15
7	Activation of the mRNA upon binding of the cap-binding complex and eIFs	5	0.005732849	0.003856728	0.289542291	4
8	Translation initiation complex formation	5	0.005637302	0.004624603	0.289542291	2
9	Translation	8	0.015096503	0.004985389	0.289542291	29
10	Formation of the ternary complex, and subsequently, the 43S complex	4	0.004968469	0.004992108	0.289542291	1

The proteomic data from MKK3^−/−^ over WT BMDM after CSE exposure was compared to generate affected pathways using REACTOME. The data is generated from 3 biological replicates.

**Table 3:** List of top affected molecules in MKK3^−/−^ BMDM.

	**MKK3^-/-^/WT: Control**		**MKK3^-/-^/WT: CSE**
**High**	Charged Multivesicular Body Protein 3	**High**	Eukaryotic translation initiation factor 4B
	Glyoxalase I		Cytochrome c oxidase subunit 4I1
	Annexin A6		GDP-mannose pyrophosphorylase B
	N-Terminal Xaa-Pro-Lys N-Methyltransferase 1		Ubiquitin fusion degradation protein UFD1
	Ceruloplasmin		Reticulocalbin 2
**Low**	SPRY domain containing 7	**Low**	Golgi transport 1B
	S100 Calcium Binding Protein A8		Capping actin protein of muscle Z-line beta subunit
	Growth Factor Independent 1 Transcriptional Repressor		Thioredoxin like 1
	Dihydrolipoamide Branched Chain Transacylase E2		Complement component 3
	Hematopoietic Prostaglandin D Synthase		Leucine-tRNA ligase

The most affected proteins, increased or decreased, in MKK3^−/−^ BMDM with and without CSE exposure were analyzed by IPA software. Benjamin-Hochberg Multiple testing correction was applied for the analysis. The data is generated from 3 biological replicates.

## 2 Experimental design, materials and methods

### 2.1 Sample preparation and iTRAQ® labeling

Cell pellets were lysed (using short 15 s sonication burst) in a RIPA buffer spiked with protease and phosphatase inhibitors. The lysates were centrifuged at 14,000 rpm for 20 min, supernatants were collected, and proteins were precipitated using chloroform:methanol:water precipitation method. The samples, three biological replicates each of the control and CSE-treated (3% vol/vol, 24 h) sample, were further processed and labeled with iTRAQ® reagents according to manufacturer׳s instructions (Sciex, Framingham, MA). Briefly, protein pellets were resuspended in 0.5 M TEAB/0.1% Rapigest buffer, reduced, alkylated, and digested with trypsin by incubating overnight at 37 °C. Protein concentration of the samples were measured by amino acid analysis of tryptic digests using Hitachi L-8900 Amino Acid Analyzer. Equal amount (15 µg) of peptides were labeled with iTRAQ® reagents, combined, desalted using MacroSpin column (The Nest Group, Inc., Southboro, MA), and dried down in a SpeedVac concentrator. Desalted labeled peptides were subsequently subjected to phosphopeptide enrichment using titanium dioxide (TiO2) resin (Glygen Corporation, Columbia, MD). The speed-vac dried flowthrough and phosphopeptide-enriched elution fractions were resuspended in buffer A (0.1% formic acid in water), and subjected to liquid chromatography-tandem mass spectrometry (LC-MS/MS) analysis.

### 2.2 Mass spectrometry data acquisition and analysis

The samples were analyzed by LC-MS/MS on a Q Exactive Plus mass spectrometer (Thermo Scientific, San Jose, CA) interfaced with nanoACQUITY UPLC System (Waters, Milford, MA) at the front end. Samples were loaded into a trapping column (nanoACQUITY UPLC Symmetry C18 Trap Column, 180 µm×20 mm, Product Number: 186006527) at a flowrate of 5 µl/min and separated with a C18 column (nanoACQUITY column Peptide BEH C18, 75 µm×250 mm, Product number: 186003545). The peptides were eluted with buffer B (0.1% formic acid in acetonitrile) gradient from 5 to 30% in 140 min at a flowrate of 300 nL/min. LC-MS/MS data were acquired using Top-20 data-dependent acquisition method. Full-scan MS spectra (m/z range 300–1700) were acquired with a resolution of 70,000, automatic gain control (AGC) target of 3e6, and a maximum injection time of 45 ms. MS/MS scans were acquired with a resolution of 17,500, AGC target of 1e5, and maximum injection time of 100 ms. The precursor ions were selected with an isolation window of 1.2 m/z and fragmented by higher collision energy dissociation (HCD) with normalized collision energies stepped to 28 and 30. Dynamic exclusion was set to 45 s to keep the repeat sequencing of peptides to minimum. Peptides and proteins were identified and quantified with Sequest HT search engine using Proteome Discoverer v2.0 (Thermo Scientific) software. A standardized iTRAQ® 8plex quantification workflow module within the Proteome Discoverer was slightly modified as below and utilized for the analysis. MS/MS data were searched against the mouse SwissProt database (downloaded in September 2015; number of protein entries=16,719). The search parameters include 10 ppm precursor mass tolerance, 0.6 Da fragment mass tolerance, and trypsin miscleavage setting of two. Static modification settings included carbamidomethylation (+57.021 Da) on cysteine and iTRAQ® 8plex (304.205 Da) on N-terminus and lysine, while dynamic modifications were set to include oxidation (+15.995 Da) on methionine and phosphorylation (+79.966 Da) on serine, threonine, and tyrosine. Peptide spectrum matches (PSMs) were verified based on q-values set to 1% false discovery rate (FDR) using the Percolator module. Reporter Ions Quantifier node was used in the processing step workflow, and the Peptide and Protein Quantifier node was used in the consensus workflow of the Proteome Discoverer v2.0 to calculate and quantify peptides and protein abundances and ratios across samples.

### 2.3 Functional annotation chart preparation

Protein with log fold change list was loaded on the IPA platform with a mouse background. The protein list was analyzed by functional annotation tool and affected pathways were analyzed. The search criteria included a minimum of 5 count and EASE score of 0.01 (Table 1).

### 2.4 Canonical pathway identification

The protein data was uploaded on IPA and REACTOME and pathway analysis was performed. The reference set was the inbuilt knowledge base, all data sources were included for analysis such as protein-protein ineteractions and microRNA-mRNA interactions. Only those relationships were considered where confidence was equal to experimentally observed relationships. Stringent criteria was applied for filetring the results. B-H multiple testing correction p-value scoring method was used and values greater than 3 with a threshold value of 0.05 are displayed.
