# Supplementary material for: Proteomics data on MAP Kinase Kinase 3 knock out bone marrow derived macrophages exposed to cigarette smoke extract
Source: Data Brief. 2017 Jun 7;13:320–5. doi: 10.1016/j.dib.2017.05.049 (PMC5476452; doi:10.1016/j.dib.2017.05.049)
Supplement: Supplementary file 1 — Supplementary material [file mmc1.docx]

Conflict of Interest: None.
